# Factors associated with pregnancy termination in women of childbearing age in 36 low-and middle-income countries

**DOI:** 10.1371/journal.pgph.0001509

**Published:** 2023-02-28

**Authors:** Djibril M. Ba, Yue Zhang, Omrana Pasha-Razzak, Chachrit Khunsriraksakul, Mamoudou Maiga, Vernon M. Chinchilli, Paddy Ssentongo

**Affiliations:** 1 Department of Public Health Sciences, The Pennsylvania State University College of Medicine, Hershey, Pennsylvania, United States of America; 2 Department of Medicine, Division of Hospital Medicine, Penn State Health Medical Center, Hershey, Pennsylvania, United States of America; 3 The Pennsylvania State University College of Medicine, Hershey, Pennsylvania, United States of America; 4 Department of Biomedical Engineering, Northwestern University, Evanston, Illinois, United States of America; 5 Department of Medicine, Penn State Health Medical Center, Hershey, Pennsylvania, United States of America; Tata Institute of Social Sciences, INDIA

## Abstract

Lack of access to safe, affordable, timely and adequate pregnancy termination care, and the stigma associated with abortion in low-middle income countries (LMICs), pose a serious risk to women’s physical and mental well-being throughout the lifespan. Factors associated with pregnancy termination and their heterogeneity across countries in LMICs previously have not been thoroughly investigated. We aim to determine the relative significance of factors associated with pregnancy termination in LMICs and its variation across countries. Analysis of cross-sectional nationally representative household surveys carried out in 36 LMICs from 2010 through 2018. The weighted population-based sample consisted of 1,236,330 women of childbearing aged 15–49 years from the Demographic and Health Surveys. The outcome of interest was self-report of having ever had a pregnancy terminated. We used multivariable logistic regression models to identify factors associated with pregnancy termination. The average pooled weighted prevalence of pregnancy termination in the present study was 13.3% (95% CI: 13.2%-13.4%), ranging from a low of 7.8 (95% CI: 7.2, 8.4%) in Namibia to 33.4% (95% CI: 32.0, 34.7%) in Pakistan. Being married showed the strongest association with pregnancy termination (adjusted OR, 2.94; 95% CI, 2.84–3.05; *P* < 0.001) compared to unmarried women. Women who had more than four children had higher odds of pregnancy termination (adjusted OR, 2.45; 95% CI, 2.33–2.56; *P* < 0.001). Moreover, increased age and having primary and secondary levels of education were associated with higher odds of pregnancy termination compared to no education. In this study, married women, having one or more living children, those of older age, and those with at least primary level of education were associated with pregnancy termination in these 36 LMICs. The findings highlighted the need of targeted public health intervention to reduce unintended pregnancies and unsafe abortions.

## Introduction

Terminating pregnancy is never an easy decision, especially in low-and middle-income countries (LMICs) where patriarchal societies, restricted abortion laws, cultural mores, religious factors, and economic factors may impact women’s decisions [1, 2]. According to a recent study, globally, between 2015 to 2019, there were an estimated 121 million unintended pregnancies per year (a rate of 64 for every 1,000 women) [3]. Pregnancy termination (PT), also known as abortion, is the removal of pregnancy tissue, products of conception or the fetus and placenta from the uterus [4]. According to the World Health Organization (WHO) women living in low-resource countries have on average more pregnancies throughout their lifetime and their risk of pregnancy-related death is much higher than those living in high-income countries (HICs) [5]. The WHO highlighted five major complications that account for nearly 75% of all pregnancy-related deaths including severe bleeding, infection, pre-eclampsia and eclampsia, complications during delivery, and unsafe abortion [5].

About 45% of all abortions are considered unsafe, of which 97% take place in LMICs [5]. The WHO defined unsafe abortion as a procedure of pregnancy termination by individuals lacking the necessary medical skills or in an environment that does not conform to minimal medical standards or both [6]. In LMICs, most abortions tend to be done clandestinely and unsafely by unskilled individuals that result in higher risk of complications such as hemorrhage, infection, uterine perforation, incomplete abortion, maternal deaths and morbidities [5, 7–9]. According to the most recent estimates, abortion rates varied gradually across World Bank income groups with the annual abortion rate of 15 abortions per 1,000 women aged 15–49 years in HICs, 44 abortions per 1,000 women age 15–49 years in middle-income countries, and 38 abortions per 1,000 women aged 15–49 years in low-income countries [3].

To accommodate strategies to reduce pregnancy termination among women of childbearing age in LMICs, it is crucial to fully understand regional and country-specific variations in the prevalence of pregnancy termination and associated factors. Such knowledge will guide the prioritization of intervention strategies to the most at-risk countries in LMICs and assist healthcare professionals and stakeholders to adequately identify potential reasons for the high prevalence of pregnancy termination. However, these estimates are lacking because most previous studies that have examined pregnancy termination in LMICs have either focused mainly on an individual country such as Ghana [10] and Ethiopia [11] or selected countries in sub-Saharan Africa (SSA) [9, 12] and yield inconsistent results using Demographic and Health Surveys data (DHS). To the best of our knowledge, there is currently no study that has examined the prevalence and factors associated with pregnancy termination in combined LMICs (SSA, North Africa, and South and Southeast Asia) and how these estimates vary across regions and countries. Thus, we aim to fill this critical knowledge gap by conducting a multi-country population-based study of the prevalence of pregnancy termination in 36 combined LMICs, mapping the geographic variations, and examining the associated socio-demographic-economic factors using the most recent Integrated Public Use Microdata Series (IPUMS) DHS data from 2010–2018. In addition to pooled analyses, we also examined country-specific factors associated with pregnancy termination.

## Methods

### Ethics

The ICF International Institutional Review Board (IRB) reviewed and approved each country’s survey. In addition, the survey also was supported by the Government of each host country. Informed written consent was obtained from all participants or their proxies. Participation was on a voluntary basis [13], and all data are entirely de-identified with no names or household addresses in the data files. Thus, no further IRB approval was needed by the authors’ institutions of the present manuscript. Detailed information on ethical matters is described elsewhere [14].

### Data source and study population

We used IPUMS DHS (https://www.idhsdata.org/idhs-action/samples) [15] to select countries in LMICs with most recent data on pregnancy termination among women of childbearing age, 15–49 years, from 2010–2018. The list of pooled 36 LMICs countries with data on pregnancy termination included in this study is reported in **Table 1**. Data collection for each host country was performed in coordination with ICF International [16]. The DHS surveys are nationally representative household surveys supported by the US Agency for International Development (USAID). The surveys employ a multistage, stratified sampling design to collect detailed information on sociodemographic characteristics, health behaviors, and reproductive health [17, 18]. The first stage involves dividing each host country into geographic regions. Within these regions, populations are stratified either by urban or rural areas. These primary sampling units (PSUs) are selected with a probability proportional to the size within each stratum. In the second stage, all households within the cluster are listed, and approximately 25 households are randomly selected for an interview using equal probability systematic sampling [19].

**Table 1:** Weighted background characteristics of the survey participants, prevalence of pregnancy termination by country and year of survey (N = 1,236,330).

			All Participants	Prevalence of Pregnancy Termination
	Survey Year	Women’s Response Rate %	N^a^ (%^b^)	% (95% CI) ^c^
**Overall**			1,236,330	13.3 (13.2, 13.4)
**SSA Countries**				
Angola	2015–2016	96.0	14,379 (1.2)	11.5 (10.5, 12.6)
Benin	2017–2018	98.0	15,928 (1.3)	11.3 (10.6, 12.0)
Burkina Faso	2010	98.4	17,084 (1.4)	11.6 (10.9, 12.2)
Burundi	2016–2017	98.8	17,269 (1.4)	12.8 (12.2, 13.5)
Cameroon	2011	97.3	15,401 (1.2)	23.6 (22.6, 24.5)
Chad	2014–2015	96.1	17,683 (1.4)	10.2 (9.5, 11.0)
Congo DR	2013–2014	98.6	18,823 (1.5)	14.6 (13.6, 15.5)
Cote d’Ivoire	2011–2012	92.7	10,052 (0.8)	18.9 (17.5, 20.3)
Ethiopia	2016	94.6	15,683 (1.3)	7.9 (7.2, 8.6)
Ghana	2014	97.3	9,396 (0.8)	24.7 (23.1, 26.3)
Guinea	2018	99.0	10,874 (0.9)	10.4 (9.6, 11.2)
Kenya	2014	96.6	14,623 (1.2)	9.3 (8.6, 10.0)
Lesotho	2014	97.1	6,621 (0.5)	10.1 (9.2, 11.1)
Liberia	2013	97.6	9,234 (0.7)	17.8 (16.1, 19.6)
Malawi	2015–2016	97.7	24,562 (2.0)	10.4 (9.8, 10.9)
Mali	2018	97.6	10,519 (0.9)	12.0 (11.1, 12.9)
Mozambique	2011	99.0	13,745 (1.1)	8.8 (8.0, 9.6)
Namibia	2013	92.3	9,173 (0.7)	7.8 (7.2, 8.4)
Niger	2012	95.4	11,159 (0.9)	15.9 (15.0, 16.9)
Nigeria	2018	99.3	41,821 (3.4)	11.7 (11.1, 12.3)
Rwanda	2014	99.5	13,497 (1.1)	12.1 (11.5, 12.7)
Senegal	2017	95.5	16,787 (1.4)	15.6 (14.7, 16.5)
South Africa	2016	86.0	8,514 (0.7)	9.3 (8.5, 10.1)
Tanzania	2015–2016	97	13,265 (1.1)	16.0 (15.3, 16.7)
Uganda	2016	97	18,506 (1.5)	18.1 (17.3, 18.8)
Zambia	2018	96.4	13,683 (1.1)	8.8 (8.1, 9.6)
Zimbabwe	2015	96.2	9,955 (0.8)	12.1 (11.3, 12.9)
**North Africa**				
Egypt	2014	99.4	21,762 (1.8)	20.8 (20.0, 21.6)
**West Asia**				
Jordan	2017–2018	97.1	14,689 (1.2)	24.2 (23.0, 25.5)
Yemen	2013	94.4	16,550 (1.3)	26.8 (25.8, 27.9)
**South & Southeast Asia**				
Afghanistan	2015	NA	29,433 (2.4)	19.5 (18.0, 20.9)
Bangladesh	2014	98.0	17,863 (1.4)	19.2 (18.4, 20.0)
India	2015–2016	96.7	699,686 (56.6)	12.0 (11.8, 12.1)
Myanmar	2015–2016	95.8	12,885 (1.0)	9.4 (8.7, 10.1)
Nepal	2016	98.3	12,862 (1.0)	20.1 (19.1, 21.1)
Pakistan	2017–2018	94.3	12,364 (1.0)	33.4 (32.0, 34.7)

Congo DR: Congo Democratic Republic

N^a^ = Weighted sample size of the combined dataset that is represented by that survey for each country

%^b^ = The % of the combined dataset represented by that survey

N^c^ = Weighted prevalence of pregnancy termination and 95% CI

The present population-based cross-sectional study included 1,236,330 women aged 15–49 years from 36 LMICs. The year of the relevant DHS survey administration for each country is displayed in **Table 1**. This study followed the Strengthening the Reporting of Observation Studies in Epidemiology (STROBE) reporting guideline [20].

### Assessment of outcome variable

The outcome variable of interest for the present study was pregnancy termination. The pregnancy termination question in the DHS included induced abortions, miscarriage, and stillbirth. Eligible survey-respondent women were asked whether they had pregnancy termination, which was measured using self-reported questionnaires such as: “have you ever had a pregnancy terminated?” The binary response of pregnancy termination (yes/no) was used as our dependent variable in a manner similar to previous publications using DHS data [9, 12].

### Assessment of explanatory variables

The following sociodemographic and socioeconomic factors available in each country were assessed to determine whether they were associated with a likelihood of pregnancy termination: country of residence, age, wealth index quintile, place of residence, educational level, marital status, frequency of reading newspaper or magazine, frequency of listening to radio, frequency of watching television, number of living children, breastfeeding status, and current contraceptive use. The aforementioned potential determinants were collected by self-report. Previous studies reported that these factors might affect women’s pregnancy termination in LMICs [9, 12]. Wealth index quintiles were determined using a principal component analysis approach of household assets (household’s ownership of several items such as television, car, radio, and other wealth-related characteristics). Detailed information on determining wealth index quintiles has been described elsewhere [21]. We also recategorized the age of participants from a continuous scale into three groups for this study (15–29, 30–39, and 40–49 years old). Marital status also was recoded into two categories (married and not married). Current contraceptive use was defined as using any methods of contraception such as oral pills, injectables, implants/Norplant, intrauterine contraceptive devices (IUDs), condoms, traditional (i.e., periodic abstinence, withdrawal and abstinence), and other methods (diaphragm, foam or jelly, other modern method) as reported in a previous study [22].

### Statistical analysis

Consistent with the DHS guidelines for analyzing the DHS data and to ensure that the estimates were nationally representative, all analyses were conducted using appropriate sampling weights, clustering, and stratification to account for the complex sampling design [23]. Univariable analyses were performed using frequency distributions for categorical variables to describe the characteristics of the study participants. The prevalence of pregnancy termination was calculated as the number of women who had pregnancy termination divided by the total number of women interviewed in that category and multiplied by 100%. Multivariable logistic regression models (proc surveylogistic; SAS institute) with appropriate sampling weight, cluster, and strata were applied to examine each independent factor’s association with pregnancy termination. In addition, to better understand country-specific differences, we also analyzed each independent factor of pregnancy termination stratified by country. Variance Inflation Factor (VIF) was calculated to measure the degree of multicollinearity among the independent variables, which did not indicate any substantial multicollinearity from the full adjusted model, with VIF values of 2 or less. Among our selected factors, 1 participant had a missing value for marital status, 33 participants for educational level, 480 participants for frequency of reading newspaper/magazine, 253 participants for frequency of listening to radio, and 527 for frequency of watching television. Considering that the proportion of missing data was very low, a complete case analysis approach was adopted. Descriptive statistics are presented as the weighted prevalence of pregnancy termination with 95% confidence intervals (CIs), and the multivariable logistic regression results are presented as adjusted odds ratios (aOR) with 95% CIs. Data were analyzed in SAS software (version 9.4-SAS Institute) at a two-tailed alpha level of 0.05 and R statistical software version 3.4.3 (R Foundation for Statistical Computing, Vienna, Austria).

## Results

### Sociodemographic characteristics of the participants

A weighted total of 1,236,330 women of childbearing age, 15–49 years, with data on pregnancy termination from 36 LMICs were included in this analysis. Most of the women were younger between ages 15–29 (53%), married (72%), not breastfeeding (80%), not currently using contraceptives (65%), had one to four children (60%), and live in the rural areas (64%). More than one-half of the women were not reading newspapers or magazines or listening to the radio. About 7% of the women were watching television at least once per week. One-half of the participants had a high school education and higher.

### Prevalence of pregnancy termination in these LMICs

The average pooled weighted prevalence of pregnancy termination in the present study was 13.3% (95% CI: 13.2%-13.4%), ranging from a low of 7.8 (95% CI: 7.2, 8.4%) in Namibia to 33.4% (95% CI: 32.0, 34.7%) in Pakistan (**Table 1**). Women aged 30–39 years, followed by women aged 40–49 years, had the highest prevalence of pregnancy termination. Women from households with the highest wealth quintile had the highest prevalence of pregnancy termination compared to other wealth categories. Furthermore, women with no education, primary education, married, living in the urban areas, not reading newspapers/magazines, listening to radio and watching television less than once a week, having more than four children, currently breastfeeding had the highest prevalence of pregnancy termination in these 36 LMICs (**Table 2**).

**Table 2:** Weighted background characteristics of the survey participants and prevalence of pregnancy termination (N = 1,236,330).

	All Participants	Prevalence of Pregnancy Termination
Characteristics	N^a^ (%^b^)	% (95% CI) ^c^
**Age groups**		
15–29	648,861 (52.5)	8.6 (8.4, 8.7)
30–39	337,449 (27.3)	18.7 (18.5, 18.9)
40–49	250,020 (20.2)	18.5 (18.2, 18.7)
**Wealth index quintile**		
Poorest	219,864 (17.8)	13.0 (12.8, 13.2)
Poorer	237,838 (19.3)	13.1 (12.9, 13.3)
Middle	248,577 (20.1)	13.0 (12.8, 13.3)
Richer	259,984 (21.0)	13.5 (13.2, 13.7)
Richest	270,068 (21.8)	13.9 (13.7, 14.2)
**Educational level**		
No education	374,322 (30.3)	15.2 (15.0, 15.4)
Primary	243,524 (19.7)	14.9 (14.6, 15.1)
Secondary	493,457 (39.9)	11.8 (11.6, 11.9)
Higher	125,002 (10.1)	11.0 (10.7, 11.2)
**Marital status**		
Not married	345,227 (27.9)	3.9 (3.8, 4.0)
Married	891,101 (72.1)	17.0 (16.8, 17.1)
**Place of residence**		
Urban	446,923 (36.1)	14.2 (14.0, 14.5)
Rural	789,406 (63.9)	12.8 (12.7, 13.0)
**Frequency of reading newspaper/magazine**		
Not at all	839,838 (68.0)	14.1 (13.9, 14.2)
Less than once a week	160,102 (13.0)	12.6 (12.4, 12.9)
At least once a week	235,937 (19.1)	11.1 (10.9, 11.4)
**Frequency of listening to radio**		
Not at all	844,640 (68.3)	13.1 (13.0, 13.3)
Less than once a week	136,039 (11.0)	13.8 (13.5, 14.1)
At least once a week	255,403 (20.7)	13.7 (13.4, 13.9)
**Frequency of watching television**		
Not at all	424,008 (34.3)	13.1 (12.9, 13.2)
Less than once a week	111,329 (9.0)	14.0 (13.7, 14.3)
At least once a week	700,481 (56.7)	13.4 (13.2, 13.5)
**Number of living children**		
None	348,014 (28.1)	4.1 (4.0, 4.2)
1–4	742,851 (60.1)	16.3 (16.1, 16.4)
More than 4	145,465 (11.8)	20.4 (20.0, 20.7)
**Breastfeeding status**		
No	993,628 (80.4)	13.2 (13.1, 13.3)
Yes	242,702 (19.6)	13.9 (13.7, 14.1)
**Contraceptive use**		
No	799,514 (64.7)	11.6 (11.5, 11.7)
Yes	436,815 (35.3)	16.5 (16.3, 16.7)

N^a^ = Weighted sample size of the combined dataset that is represented by that survey for each country

%^b^ = The % of the combined dataset

N^c^ = Weighted prevalence of pregnancy termination and 95% CI

ref = reference

### Pooled analyses of factors associated with pregnancy termination in LMICs

**Fig 1:**
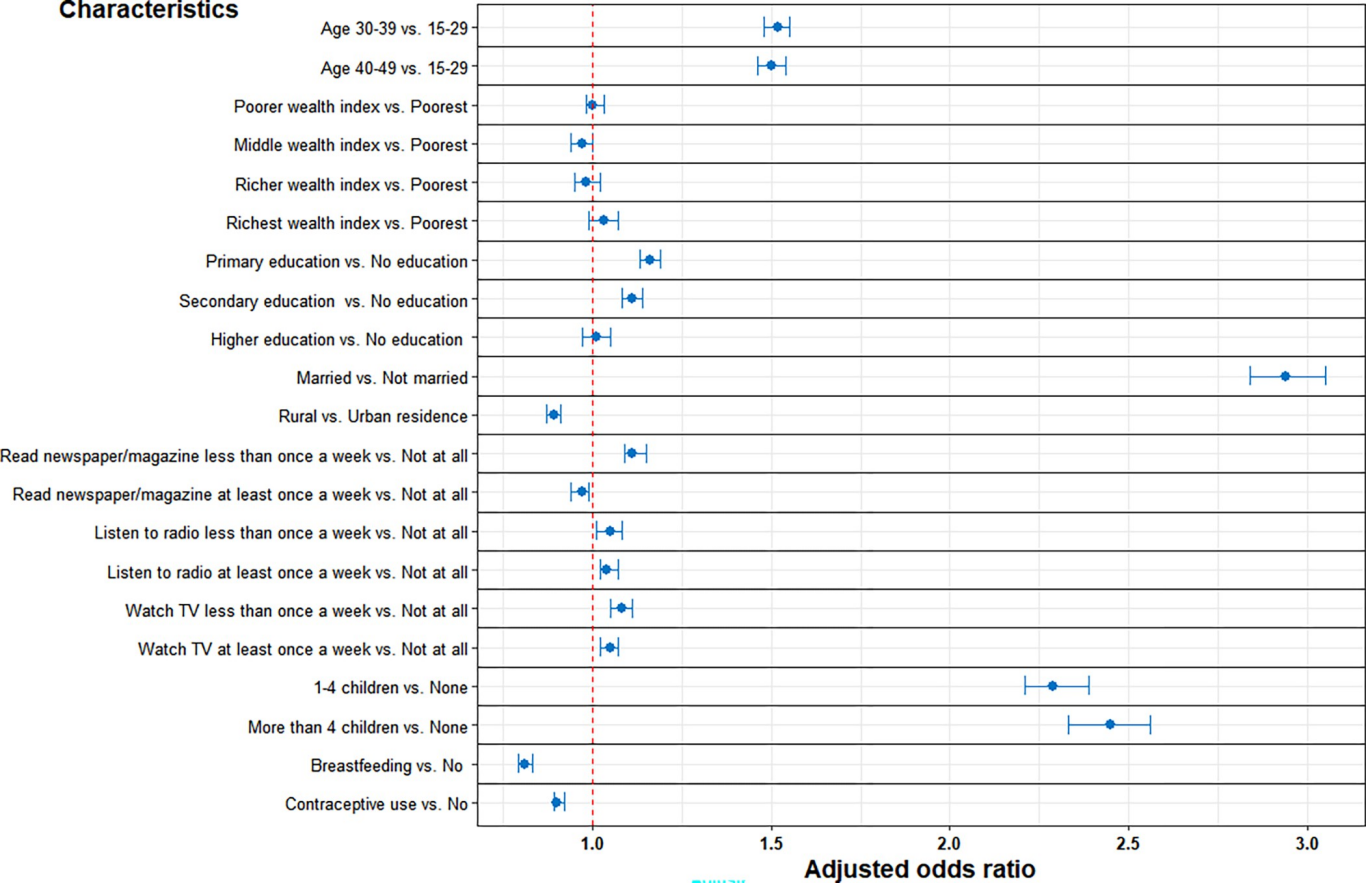
Adjusted odds ratio and 95% confidence limits. Independent factors associated with pregnancy termination in 36 LMCIs.

The full multivariable regression results from the pooled analyses are shown in **Table 3, Fig 1**. In the fully adjusted model, except for wealth index, all the remaining factors were significantly associated with the odds of pregnancy termination. Married women displayed the strongest association with pregnancy termination (adjusted OR, 2.94; 95% CI, 2.84–3.05; *P* < 0.001) compared to unmarried women. Women who had more than four children had higher odds of pregnancy termination (adjusted OR, 2.45; 95% CI, 2.33–2.56; *P* < 0.001), followed by women with one to four children compared to those without children. Women aged 30–39 had higher odds of pregnancy termination (adjusted OR, 1.52; 95% CI, 1.48–1.55; *P* < 0.001), followed by women aged 40–49 (adjusted OR, 1.50; 95% CI, 1.46–1.54; *P* < 0.001) compared to women aged 15–29. Moreover, women who had primary and secondary levels of education had higher odds of pregnancy termination compared to those with no education. Interestingly, we did not observe a significant association between the household wealth index and the odds of pregnancy termination. Additionally, women who read newspapers/magazines less than once a week, watch television, and listen to radio more frequently had higher odds of pregnancy termination compared to those who did not all. Additionally, women currently breastfeeding and using contraceptives had lower odds of pregnancy termination.

**Table 3:** Multivariable logistic regression model showing odds ratios (95% confidence intervals) for the sociodemographic-economic factors of pregnancy termination.

Characteristics	Odds Ratios	(95% CI)	*P*-value
**Age groups**			
15–29	1 (reference)		
30–39	1.52	(1.48, 1.55)	<0.001
40–49	1.50	(1.46, 1.54)	<0.001
**Wealth index status**			
Poorest	1 (reference)		
Poorer	1.00	(0.98, 1.03)	0.79
Middle	0.97	(0.94, 1.00)	0.10
Richer	0.98	(0.95, 1.02)	0.29
Richest	1.03	(0.99, 1.07)	0.09
**Education**			
No education	1 (reference)		
Primary	1.16	(1.13, 1.19)	<0.001
Secondary	1.11	(1.08, 1.14)	<0.001
Higher	1.01	(0.97, 1.05)	0.81
**Marital status**			
Not married	1 (reference)		
Married	2.94	(2.84, 3.05)	<0.001
**Place of residence**			
Urban	1 (reference)		
Rural	0.89	(0.87, 0.91)	<0.001
**Frequency of reading newspaper/magazine**			
Not at all	1 (reference)		
Less than once a week	1.11	(1.09, 1.15)	<0.001
At least once a week	0.97	(0.94, 0.99)	0.02
**Frequency of listening to radio**			
Not at all	1 (reference)		
Less than once a week	1.05	(1.01, 1.08)	0.001
At least once a week	1.04	(1.02, 1.07)	0.002
**Frequency of watching television**			
Not at all	1 (reference)		
Less than once a week	1.08	(1.05, 1.11)	<0.001
At least once a week	1.05	(1.02, 1.07)	0.003
**Number of living children**			
None	1 (reference)		
1–4	2.29	(2.21, 2.39)	<0.001
More than 4	2.45	(2.33, 2.56)	<0.001
**Breastfeeding status**			
No	1 (reference)		
Yes	0.81	(0.79, 0.83)	<0.001
**Contraceptive use**			
No	1 (reference)		
Yes	0.90	(0.89, 0.92)	<0.001

**Model** fully adjusted for country of residence, age, education status (categorical), marital status (married/not married), wealth index status (categorical), place of residence (urban/rural), frequency of reading newspaper/magazine (categorical), frequency of listening to radio (categorical), frequency of watching television (categorical), number of living children (categorical), breastfeeding status (yes/no), contraceptive use (yes/no).

### Country-specific analyses

**Fig 2:**
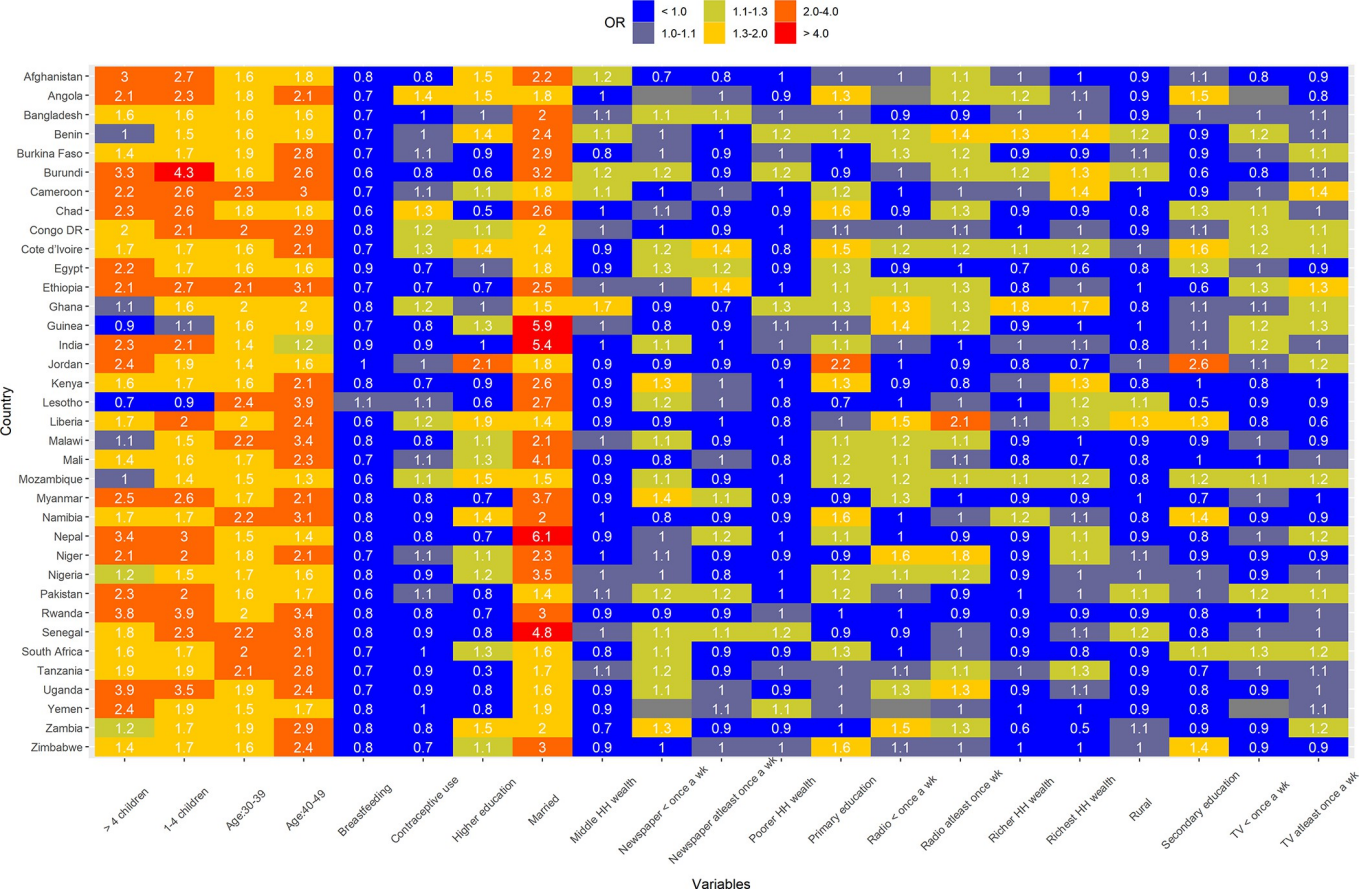
Country-specific adjusted odds ratios for factors associated with pregnancy termination in 36 LMICs.

Married women had the strongest association with pregnancy termination, with ORs above 1.30 for all countries (**Fig 2**). The magnitudes of ORs for married women ranged from 1.37 (95% CI, 1.12–1.67) in Liberia to 6.12 (95% CI, 4.22–8.88) in Nepal. Marital status was followed by older age women, which was consistently associated with pregnancy termination, with ORs above 1.10 for all countries (**Fig 2**). The magnitudes of ORs for aged 40–49 ranged from 1.18 (95% CI, 1.14–1.23) in India to 3.87 (95% CI, 2.79–5.35) in Lesotho. A similar significant association also was observed for women aged 30–39. Although, we did not observe a significant association between wealth index and pregnancy termination in the pooled analysis, the magnitude of association in Ghana, Benin, Cameroon, Burundi, and India were substantial with ORs above 1. Women with one or more living children were consistently strongly associated with higher odds of pregnancy termination in all countries except Lesotho and Guinea. Importantly, breastfeeding was consistently associated with lower odds of pregnancy termination in all countries except Lesotho, Kenya, Namibia, Egypt, and Jordan. The magnitudes of ORs for other factors were very heterogeneous (**Fig 2**).

## Discussion

In this cross-sectional study of more than 1 million women of childbearing age, 15–49 years, from 36 LMICs, our pooled results showed that the mean weighted prevalence of pregnancy termination was 13% and exhibited substantial between-country variation. Married women, having one or more children, of older age, having a primary and secondary levels of education, exposure to mass media, breastfeeding, and use of contraceptives were significantly associated with pregnancy termination in these 36 LMICs. Interestingly, we did not observe a significant association between the household wealth index and the odds of pregnancy termination. The pooled results indicated that Pakistan—a South Asian country—had the highest prevalence of pregnancy termination and Namibia—a SSA country—had the lowest prevalence. Being married and increasing age were the leading factors associated with pregnancy termination in the pooled analysis. The relative magnitude of other factors showed considerable heterogeneity among countries.

Our findings indicate that being married and older are some of the major factors associated with pregnancy termination in these 36 LMICs, and are generally consistent with the results of prior studies [10, 12, 24]. Advanced maternal age women are more vulnerable to a high-risk pregnancy including maternal health problems and pregnancy related complications such as preeclampsia, ectopic pregnancy, and gestational diabetes, which may lead to miscarriages or stillbirths [25–27]. The positive association between married women and the higher odds of pregnancy termination could be attributed to the lack of contraceptive use or contraceptives’ failure [28]. Married women from low socio-economic status may not have the financial means to pay for contraceptives, which may result in an increased likelihood of unintended pregnancies and maternal mortality rates due to unsafe abortions [22].

Our findings of positive associations between primary and secondary education levels and the higher odds of pregnancy termination compared to women with no education are consistent with previous studies [9, 29]. A plausible explanation could be due to the fact that educated women, especially in low resource settings, may have pregnancies that could hinder with their education and therefore may decide to terminate those pregnancies [9].

Consistent with previous studies, we found that women who are exposed to mass media**—**television and radio—were more likely to terminate pregnancies compared to those who did not [9, 24]. Exposure to mass media is associated with self-efficacy in abortion decision-making among adolescent girls and young women in LMICs [30]. More importantly, we observed a negative association between contraceptive use and breastfeeding and the odds of pregnancy termination. Such findings could be attributed to a perfect use of contraceptive methods and lactational amenorrhea method and highlight the need to promote and increase the uptake of contraceptive use in preventing unintended pregnancies in low resource areas.

### Public health relevance and recommendation

Our current analysis suggests that policies that will effectively reduce the prevalence of pregnancy termination should target individuals such as married women, older women, those with frequent exposure to mass media, increase number of living children, and women who were not using any birth control methods. Additionally, public health interventions working with sectors such as education, radio, and television stations to promote health education about the benefits of contraceptive use, especially among older and married women, are critically needed. Targeting these individuals before becoming pregnant may significantly lower the risk of unintended pregnancies and save life. More importantly, providing birth controls assistance at no costs and promoting breastfeeding may also play an essential role in reducing the risk of unintended pregnancies and unsafe abortions in these LMICs.

### Study strengths and limitations

Our study has major strengths. This is one of the few comprehensive studies to investigate the prevalence and the factors associated with pregnancy termination using the most recent DHS data across a nationally representative collection of multiple LMICs with large sample size and high response rate. Notwithstanding, interpretation of our findings should be done in light of the study’s limitations as there are several measurement issues related to self-reports abortion survey data. First, pregnancy termination was not stratified into intended abortion, miscarriages, and stillbirths due to the lack of these data from DHS. Due to a lack of stratified analysis, we are limited in advocating termination-specific public health interventions. Future DHS endeavors should strive to separate the pregnancy termination questions to capture distinct responses. Second, the cross-sectional nature of the survey does not allow for determining causality. Third, women tend to under-report when they had illegal induced abortion. One potential explanation is that women may be reluctant to admit to an illegal act and may be more reluctant than others to participate in an abortion related survey, especially in LMICs, where social and cultural norms play a significant role in women’s decision [31]. Therefore, when interacting with survey interviewers, many women may have kept secret any information related to their pregnancy termination in the past to preserve their social image, which may under-represent true pregnancy termination rate [31, 32]. Fourth, the pregnancy termination questionnaire was self-reported and could be subject to recall bias, which may have also contributed to underreporting of pregnancy termination. Regardless of these limitations, the self-report technique of abortions provides a more solid empirical picture for the estimation of pregnancy termination in LMICs than does a reliance on the opinions of healthcare professionals [31]. Lastly, the year of data widely vary between countries from 2010–2018, which is due to the availability of data that were collected by the DHS program for each country included in this analysis. To the best of our knowledge, this is the first comprehensive study to estimate prevalence pregnancy termination, associated factors and country-level variations among women of childbearing age in 36 LMICs.

## Conclusions

Findings from this study indicate great heterogeneity in the prevalence of pregnancy termination across these 36 LMICs and are related to women’s demographic and personal characteristics. Married women, having one or more living children, older age women, and those with primary and secondary levels of education were the leading factors associated with pregnancy termination. We highlight the need for future demographic and health surveys to distinguish between induced abortion, miscarriages, and stillbirths. Such a distinction will guide pregnancy termination-specific public health and medical intervention needs.
